# Analysis of Polyhydroxyalkanoates Granules in *Haloferax mediterranei* by Double-Fluorescence Staining with Nile Red and SYBR Green by Confocal Fluorescence Microscopy

**DOI:** 10.3390/polym13101582

**Published:** 2021-05-14

**Authors:** Verónica Cánovas, Salvador Garcia-Chumillas, Fuensanta Monzó, Lorena Simó-Cabrera, Carmen Fernández-Ayuso, Carmen Pire, Rosa María Martínez-Espinosa

**Affiliations:** 1 Technological Centre of Footwear and Plastic of the Region of Murcia (CETEC) Avda, Europa 4-5, 30840 Alhama de Murcia, Spain; schumillas@gmail.com (S.G.-C.); f.monzo@ctcalzado.org (F.M.); c.fernandez@ctcalzado.org (C.F.-A.); 2 Cetec Biotechnology, Avda, Europa 4-5, 30840 Alhama de Murcia, Spain; 3 Department of Agrochemistry and Biochemistry, Biochemistry and Molecular Biology Division, Faculty of Science, University of Alicante, Carretera San Vicente del Raspeig s/n, San Vicente del Raspeig, 03690 Alicante, Spain; lorena.simo@ua.es (L.S.-C.); carmen.pire@ua.es (C.P.); 4 Multidisciplinary Institute for Environmental Studies “Ramón Margalef”, University of Alicante, Ap. 99, 03080 Alicante, Spain

**Keywords:** haloarchaea, *Haloferax mediterranei*, polyhydroxyalkanoates (PHAs), Nile red, SYBR Green, confocal microscopy, transmission electronic microscopy

## Abstract

*Haloferax* *mediterranei* is a haloarchaeon of high interest in biotechnology because it produces and mobilizes intracellular polyhydroxyalkanoate (PHA) granules during growth under stress conditions (limitation of phosphorous in the culture media), among other interesting metabolites (enzymes, carotenoids, etc.). The capability of PHA production by microbes can be monitored with the use of staining-based methods. However, the staining of haloarchaea cells is a challenging task; firstly, due to the high ionic strength of the medium, which is inappropriate for most of dyes, and secondly, due to the low permeability of the haloarchaea S-layer to macromolecules. In this work, *Haloferax mediterranei* is used as a halophilic archaeon model to describe an optimized protocol for the visualization and analysis of intracellular PHA granules in living cells. The method is based on double-fluorescence staining using Nile red and SYBR Green by confocal fluorescence microscopy. Thanks to this method, the capability of PHA production by new haloarchaea isolates could be easily monitored.

## 1. Introduction

Polyhydroxyalkanoates (PHAs) are biopolymers produced and accumulated in many prokaryotic microbes (bacteria or archaea) as carbon and energy storage materials, ensuring their survival under stress conditions [1]. Typically, PHA biosynthesis is favored by limiting an essential nutrient—such as nitrogen, phosphorous, or oxygen—for microbial growth, with a parallel high availability of an exogenous carbon source [2]. They are water insoluble polymers and stored in the cell cytoplasm as granules. PHAs are biodegradable, biocompatible, and thermoplastic polymers, and due to these features, they have recently attracted increasing attention for their industrial application in the production of biodegradable plastic used for packaging or in the biomedical field [3,4].

In this context, extremophilic microbes in general, and particularly the haloarchaea group (Archaea domain), have attracted the attention of the scientific community due to their peculiar metabolic capabilities. In addition to the applicability in bioplastic production shown by some species, their adaptations to extreme environments make the haloarchaea a promising source of different biocompounds of high interest in biotechnology and biomedical applications [5,6]. For instance, carotenoids such as bacterioruberin and lycopene with a high antioxidant activity, as well as extracellular polymeric substances (EPS), mainly composed of polysaccharides and exhibiting high viscosity, are suitable natural additives in the food, pharmaceutical, and cosmetic industries [7]. Moreover, other biocompounds and biomolecules of significant interest are gas vesicles, which are protein-based buoyancy organelles produced by some halophilic archaea with a potential role in immunology for the generation of vaccines due to their role as scaffolds during the presentation of epitopes [6]; ether-linked lipids as novel drug delivery systems [8]; bacteriorhodopsin, a photochemical material for bioelectronics and photochemical processes [5]; and enzymes with high activity and stability at high temperatures, and high ionic strength in industrial applications [9].

*Haloferax mediterranei* is one of the better-described haloarchaea at the time of writing this work. It is an extreme halophilic capable of accumulating poly(3-hydroxybutyrate-co-3-hydroxyvalerate) (PHBV) from many cheap carbon sources [10,11,12,13,14]. The key enzyme involved in the biosynthesis of PHA in haloarchaea is PHA synthase, which catalyses the polymerization of the hydroxyalkanoate monomer to produce PHA chains. PHA synthase in haloarchaea consists of two subunits, PhaC and PhaE, and belongs to class III [15,16,17]. Apart from this enzyme, two other proteins are essential for PHA synthesis in haloarchaea—a 3-ketoacyl-acyl carrier protein reductase (PhaB) and a structural protein called PHA granule-associated protein (PhaP), which is involved in polyhydroxyalkanoate accumulation and granule formation [18]. The main advantage of using haloarchaea as cell factories to produce PHAs as compared to bacteria is that haloarchaeal cultures on a moderate or large scale do not need strict sterile conditions due to the high concentrations of salt needed by the cells to stay alive. This makes cultivation easier and more convenient as compared to eubacterial strains. Besides, raw materials like brines could even be used to prepare the culture media for these strains, thus contributing to the circular economy processes [7,19]. Hence, haloarchaea are revealed as a promising natural source for bioplastic production in an attempt to reduce global contamination caused by plastic production and microplastic accumulation [20,21].

Different approaches have been used for the screening of PHA-producing microbes and/or the imaging of PHA granules [22,23,24,25,26]. The main aim of these approaches is to quickly identify, at low cost and from a biotechnological point of view, the capability of new isolates of microbes of high interest to produce PHAs. From among those approaches, transmission electron microscopy (TEM) is an excellent tool for imaging PHA granules [27]. However, it is not useful for screening living PHA-producing cells. In addition, it is a labor-intensive, expensive, and time-consuming technique that needs highly experienced and skilled staff. Easy and rapid approaches based on cellular staining can be used to determine whether the cells are able to produce PHAs. Most extended protocols in bacteria include the use of lipophilic dyes such as Sudan black B, Nile blue A, and Nile red [23,25,28,29]. While the staining of cells with Sudan black B and Nile blue A requires heat-fixed cells, Nile red (which is a highly fluorescent and photostable organic dye with a greater affinity for PHAs than Sudan black B) allows the staining of living cells under growing conditions [24,26]. Due to their lipophilic nature, Nile blue A and Nile red staining are very useful for visualizing PHA granules, which are surrounded by a lipid membrane, thus appearing as bright fluorescent red granules [24,30]. Nile red has been widely used for the in vivo screening and identification of PHA-producing bacteria on agar plates through the observation of fluorescent colonies under UV light [23,24,26]. However, due to the lipophilic nature of Nile red and Nile blue A, they can occasionally bind unspecifically to other lipid inclusions, membranes, or cell envelopes and can lead to ambiguous results [31,32]. For this reason, staining with an additional fluorescence dye can be appropriate to co-localize PHA granules.

Fluorescence staining in halophilic microbes can be challenging. Firstly, this is due to the high ionic strength of the medium, which can be inappropriate for most of dyes (the removal of salt crystals is often difficult or even impossible, thus negatively affecting the final quality of the images); secondly, particular characteristics of archaea, including differences in cell membrane lipids and cell walls may hinder staining [33,34]. In contrast to bacteria, all archaea possess cell walls lacking peptidoglycan, the glycerol linkage between the phospholipid head, and the side chain is in the L-isomeric form instead of the D-isomeric form; furthermore, membrane lipids present ether linkages as opposed to the ester-linked lipids found in bacteria and eukaryotes, thus providing more chemical stability to the membrane [34,35]. Moreover, a paracrystalline protein surface layer (S-layer) is generally present in nearly all archaea, reducing the permeability to macromolecules [33]. These limitations can explain the scarce number of works reporting the use of Nile red staining in haloarchaea for the visualization of PHA granules [31]. In this work, a rapid and easy method based on a double-fluorescence staining method is described for the first time in its identification and analysis of PHA granules in unfixed *Hfx. mediterranei* cells with the use of confocal microscopy. SYBR Green is a green fluorescent cyanine dye that has a high affinity for double-stranded DNA, but also for single-stranded DNA and RNA, although with less intensity [36,37]. The mode of binding is believed to be a combination of DNA intercalation and external binding. In this article, we show that double-staining with SYBR Green I and Nile red is compatible with high ionic-strength media and is useful for co-localizing PHA granules within the cytoplasm of living haloarchaea cells, thus improving the analysis of the granules (size and shapes) and their location within the cytoplasm.

## 2. Materials and Methods

### 2.1. Cell Culture

The *Haloferax mediterranei* strain R-4 (ATCC33500), previously isolated by Rodríguez-Valera et al. (1980) from saltern ponds located in Santa Pola, Alicante, Spain, was used for all experiments [38]. The growth medium was adapted from Lillo and Rodriguez-Valera (1990) [39] and contained (g/L) (minimal medium): 194.9 g NaCl; 49.4 g MgSO_4_·7H_2_O; 34.6 g MgCl_2_·6H_2_O; 5.0 g KCl; 0.17 g NaHCO_3_; 0.58 g NaBr; 0.92 g CaCl_2_; 0.005 g FeCl_3;_ 1 g KNO_3_ as nitrogen source; 0.25 g NaH_2_PO_4_ as phosphorus source; and 10 g of glucose as carbon source (PanReac AppliChem, Darmstadt, Germany) at pH 7.2 [16]. The pH level was first adjusted with NaOH 1N. *Hfx. mediterranei* cells were diluted in a growth medium to an initial optical density of approximately 0.20 at 600 nm (OD_600nm_) (with growth medium used for blank). An amount of 100 mL of cell cultures was developed in 500 mL flasks at 42 °C, in a horizontal incubator (BioSan, Riga, Latvia) with a shaking platform (170 rpm).

### 2.2. Sample Preparation and Fluorescence Staining

After approximately 72 h of incubation, 10 mL of cell culture at the late exponential growth phase (OD_600nm_ = 1.50) were transferred to a new 50 mL flask and used for Nile red staining. The other 90 mL of the cell culture were kept in incubation for further TEM analysis and PHA extraction. Nile red (Sigma-Aldrich, Darmstadt, Germany, cat. number N3013) was dissolved in methanol to obtain a 500 µg/mL stock solution. The solution was stored at 4 °C for 1 month. To stain *Hfx. mediterranei* cells, the Nile red stock solution was added to the 10 mL of the growth medium at a 1:1000 dilution (to a final concentration of 0.5 µg/mL) and cells were cultured in a 50 mL flask for 24 h at 42 °C, under shaking conditions (170 rpm). After this incubation time (OD_600nm_ = 1.95), the cells stained with Nile red were harvested by centrifugation at 3000× *g* for 15 min and washed twice with a saline buffer (10% NaCl, 0.1 M sodium phosphate buffer, pH 7.2). The pellet was resuspended in 100 µL of the saline buffer and 2 µL of SYBR Green (dsGreen 100X qPCR, Canvax Biotech, Córdoba, Spain) were added to the 1.5 mL Eppendorf tube containing Nile red-stained cells, mixed by vortexing and incubated in the dark at room temperature for 15 min. The cells were washed with the saline buffer, collected by centrifugation at 3000× *g* for 15 min, and resuspended in 100 µL of the saline buffer for further confocal microscopy analysis.

### 2.3. Confocal Fluorescence Microscopy

For fluorescence and phase contrast microscopy, a drop of the previous sample (stained cells resuspended in saline buffer) was pipetted in a slide. Then, cells were covered with a cover slip and edges of the cover slip were sealed with nail polish. The cells were imaged with a confocal microscope Leica *TCS SP8* (Leica Microsystems, Wetzlar, Germany) using an HC PL APO CS2 63x/1.3 Glycerol objective, in a 1024 × 1024 format, at 400 Hz scan speed and hybrid detectors (HyD). The cells were excited by 553 nm Ar-laser for Nile red and by 497 nm Ar-laser for SYBR Green and detected with a Leica HyD hybrid detector (569nm–648nm). All images were processed using LAS X 3.5.5.19976 (Leica) and ImageJ Fiji vl.50c.

### 2.4. Transmission Electron Microscopy (TEM)

After 96 h, 20 mL of the cell culture in the stationary phase (OD_600nm_ = 1.95) were harvested by centrifugation at 3000× *g* for 15 min for the TEM analysis. The cells were washed twice with a saline buffer (10% NaCl, 0.1M sodium phosphate buffer, pH 7.2), resuspended in a 2.5% (*v*/*v*) glutaraldehyde solution prepared in saline buffer for primary fixation overnight at 4 °C. Following the primary fixation, the cells were pelleted and washed three times with a saline buffer. The cell pellets were then fixed with 1.0% osmium tetroxide in SP buffer (1% *v*/*v*) for 2 h at 4 °C and subsequently washed three times with a saline buffer. A third fixative solution was added to the cells, 0.5% (*w*/*v*) uranyl acetate solution (in Veronal-acetate buffer) and cells were incubated overnight in the dark at 4 °C. The ensuing steps were performed according to the procedure described by Tian et al. (2005) [40]. Photomicrographs were taken with a Philips Tecnai 12 Electron Microscope (Carl Zeiss, Oberkochen, Germany), capable of imaging negatively stained samples at voltages ranging between 80 Kv and 120 Kv, equipped with a megaview III digital camera.

### 2.5. Determination of Cell Dry Weight (CDW) and PHBV Extraction and Purification

After fermentation, the rest of the cell culture (approximately 70 mL) was harvested by centrifugation at 3000× *g* for 15 min. Then, the cells were washed with a 10% *w*/*v* NaCl solution and were centrifuged again to remove debris. The cell pellet was resuspended in deionized water with 0.1% sodium dodecyl sulfate (SDS) to achieve hypo-osmotic shock and complete cells lysis and dried at 70 °C for at least 5 h and weighed to determine its CDW. The dry pellet was treated with hot chloroform at 90 °C for 6 h, followed by precipitation with 10 volumes of pre-chilled methanol. To collect PHAs, the precipitate was centrifuged at 4000× *g* for 25 min and PHAs were dried at 70 °C for 2 h to remove all residual solvent. The pellet, corresponding to raw PHBV, was recovered, and weighed. The polymer yield was calculated as [PHBV (g)/CDW (g)].

### 2.6. Attenuated Total Reflectance–Fourier Transform Infrared (ATR–FTIR) Analysis

Aliquots of the polymer were subjected to the ATR-FTIR analysis. The spectrum was recorded using a Spectrum Two FT-IR Spectrometer (Bruker Vertex 70–80, Billerica, MA, USA) from 400 to 4000 cm^−1^, with a resolution of 4 cm^−1^ and averaged over 32 scans.

### 2.7. Statistics

For the analysis of PHA granule size, the Image J Fiji free software was used to measure the diameter of PHA granules. Analysis of data was performed using GraphPad Prism 5 software and a *p*-value < 0.05 was taken as the level of significance. The data are the means of three independent experiments ± standard deviations (n = 3).

## 3. Results

### 3.1. Nile Red Staining and Detection of PHA Granules by Confocal Microscopy

**Figure 1:**
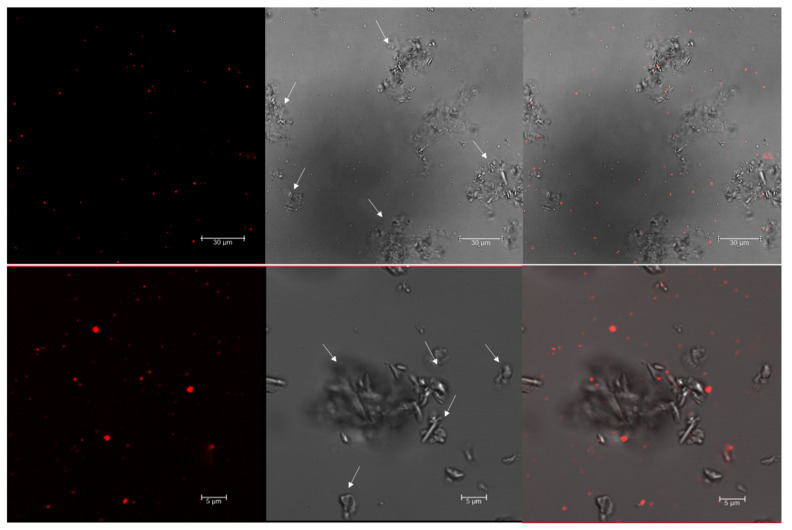
Confocal images of Nile red-stained *Haloferax mediterranei* cells. The *Hfx. mediterranei* cells in the stationary phase were stained with Nile red (0.5 µg/mL) and imaged with a confocal microscope 4 days after subculture. Arrows point to crystals of mineral salts. From left to right: red fluorescence PHA granules; phase contrast image; overlay image of red fluorescence and phase contrast image.

Staining with Nile red was performed with the cells of a known producer of PHBV, *Haloferax mediterranei*, growing in an aqueous and high-salt-grown medium (3.3 M NaCl). Confocal fluorescence microscopy, at an excitation wavelength of 553 nm, revealed numerous brightly fluorescent red granules within the cells corresponding to PHA granules (Figure 1). However, the identification of cells by phase contrast microscopy for the localization of PHA granules inside the cells [26,31,41,42] is not a suitable technique for haloarchaea due to the high concentration of salts in the culture medium as well as in the cytoplasm, making the image quality not optimal due to the presence of salt crystals (Figure 1).

**Figure 2:**
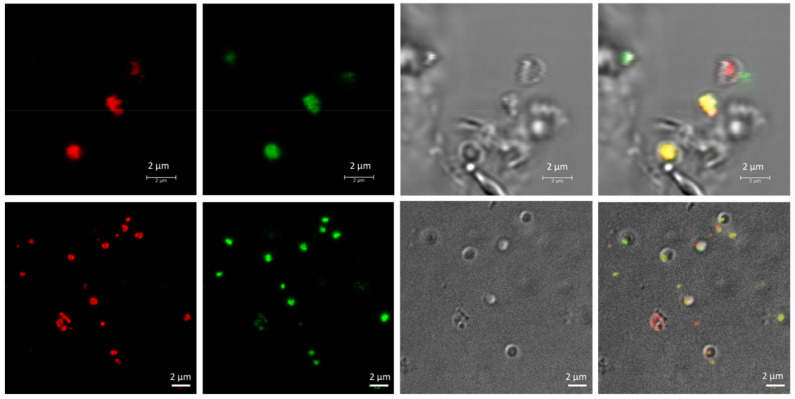
Fluorescence confocal micrographs of living *Haloferax mediterranei* cells, with PHA granules stained with Nile red and SYBR Green. Unfixed *Hfx. mediterranei* cells were co-stained with Nile red lipid fluorescence dye and the nucleic acid-staining dye SYBR Green, revealing the intracellular co-localization of PHA granules with numerous DNA spots in the cytoplasm of the cell, as visualized during confocal microscopy. From left to right: Nile red, SYBR Green, phase contrast and merged channels of Nile red, SYBR Green, and phase contrast images.

**Figure 3:**
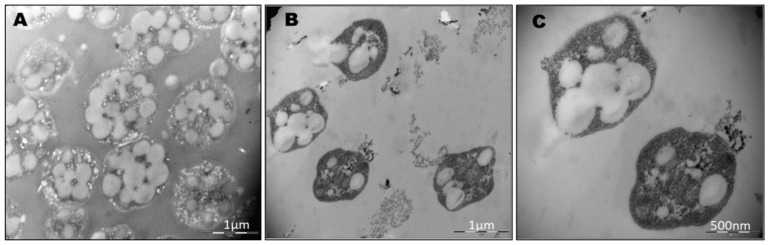
Electron micrograph of ultra-thin sections of Haloferax mediterranei. (**A**), (**B**), and (**C**) show electron micrographs of Hfx. mediterranei cells demonstrating the accumulation of PHA granules. Cells were cultured at 42 °C for 96 h in a phosphorous-limited medium.

To avoid interference in the identification of the cells and the co-localization of the PHA granules inside the cells due to the presence of salt crystals on microscopy, the cells were stained using Nile red and SYBR Green dyes (as described in Materials and Methods) and observed using confocal fluorescence microscopy (Figure 2). As mentioned above, fluorescence microscopy at an excitation wavelength of 553 nm revealed numerous brightly fluorescent red granules within the cells, corresponding to PHA granules. An excitation wavelength of 497 nm revealed fluorescent green spots corresponding to stained DNA in the cytoplasm of cells. Merged images confirmed the intracellular co-localization of PHA granules in the cytoplasm of cells, which would otherwise be complicated due to the presence of salts in the medium masking cells and low-resolution contrast phase images (Figure 2). TEM pictures of the same culture confirmed the presence of PHA granules (Figure 3).

### 3.2. Analysis of PHA Granules Size

**Figure 4:**
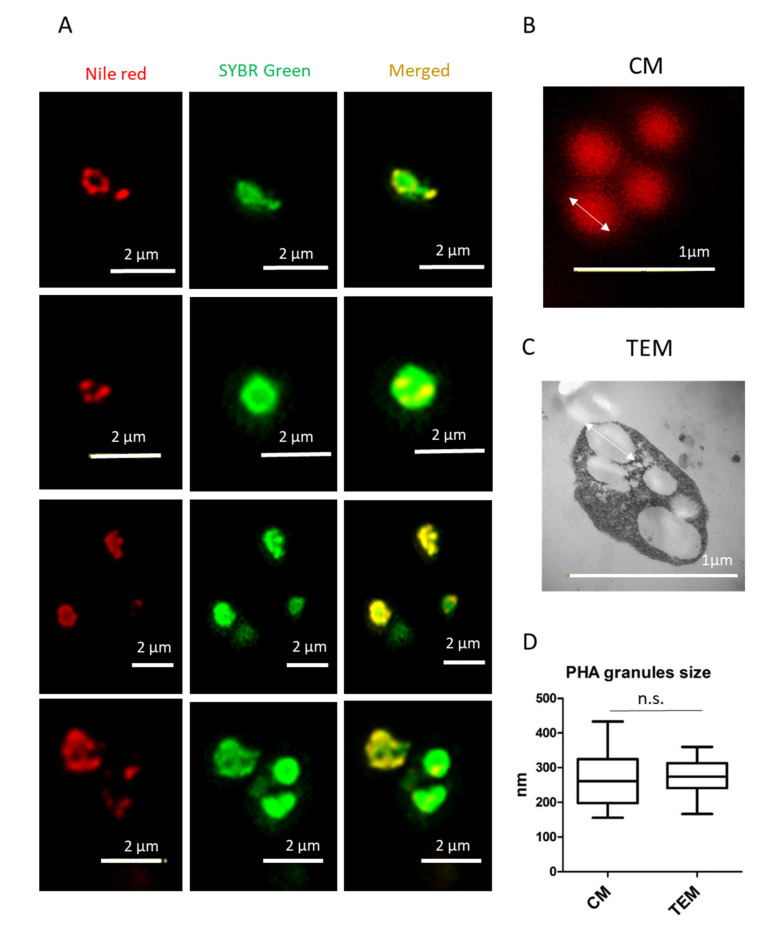
Analysis of PHA granule size stained with Nile red by confocal microscopy in living *Haloferax mediterranei* cells. (**A**) Unfixed *Hfx. mediterranei* cells were co-stained with Nile red lipid fluorescence dye and the nucleic acid-staining dye SYBR Green, revealing the intracellular co-localization of PHA granules with numerous DNA spots in the cytoplasm of the cell as visualized during confocal microscopy. From left to right: Nile red, SYBR Green, and merged channels of Nile red and SYBR Green. (**B**) Image of PHA granules taken by confocal microscopy (CM). (**C**) Image of PHA granules taken by transmission electron microscopy (TEM). (**D**) Box and whisker plot represents PHA granule size (CM n = 14 and TEM n = 16). Image J software was used to measure PHA granule size, and data were plotted and analyzed with GraphPad Prism 5 software. Non-significant differences in granule size were found after comparing the two methods (*p* = 0.979).

Double staining with Nile red and SYBR Green allows the visualization of PHA granules in high resolution and the precise co-localization of PHA granules in the cytoplasm of *Hfx. mediterranei* cells (Figure 4A). The size of the PHA granules ranged from approximately 150 to 450 nm, as analyzed in the confocal images (Figure 4B). Similar results were obtained when TEM micrographs were used to analyze PHA granule size (Figure 4C,D), confirming that fluorescence co-staining of *Hfx. mediterranei* cells with Nile red and SYBR Green can be a useful and rapid approach in visualizing and analyzing PHA granule size.

### 3.3. PHA Extraction and Polymer Characterization by ATR–FTIR Spectroscopy

The capabilities of *Hfx. mediterranei* to accumulate PHAs were evaluated at the shaking flask level in a phosphorous-limited medium with 10 g/L glucose. After 96 h of incubation at 42 °C, the PHBV synthesis corresponded to 0.198 ± 0.06 g/L (Table 1). The polymer extraction confirms the presence of PHA granules.

**Figure 5:**
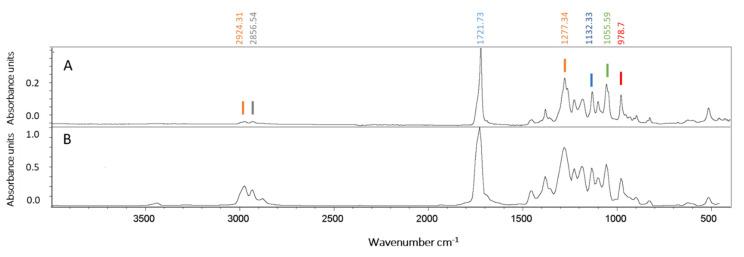
Polymer characterization by ATR-FTIR spectroscopy. Attenuated total reflectance-Fourier transform infrared (ATR-FTIR) spectra of the PHBV purified from *Hfx. mediterranei* (panel **A**) and of the commercial reference Sigma Aldrich PHBV with a PHV content of 12 mol% (cas number 80181-31-3) (panel **B**).

The purified polymer was analyzed by the FTIR spectrophotometer with ATR and compared with a commercial PHBV (Figure 5). The FTIR spectra of both samples showed an absorption peak near 1721 cm^−1^, which corresponds to the ester carbonyl bond (C=O), the most important feature of the copolymer 3-hydroxybutyrate-*co*-3-hydroxyvalerate (PHBV) [43]. Other absorption bands for the polymer sample obtained under the conditions of this study (Figure 5) were found in the range 2924–2856 cm^−1^ (CH, CH_2_ symmetric and asymmetric stretching), 1450–1380 cm^−1^ (C-C stretching), at 1132 cm^−1^ (C-O stretching), and in the range 978–821 cm^−1^ corresponding to C-C deformation [10]. The spectra corresponded to the typical profile of a copolymer PHBV, previously reported in *Hfx. mediterranei* [16].

## 4. Discussion

As highlighted in the introduction of this article, haloarchaea have emerged as promising and valuable biotechnological platforms for the production of compounds of high interest in the industry; these are compounds such as carotenoids, EPS, ether-linked lipids, and PHAs amongst others [6,7,13]. Haloarchaeal cultivation is easier and more convenient compared to eubacterial strains—its low requirement for sterile conditions results from the high concentrations of salt needed by the cells to stay alive [10,12,14]. Furthermore, cell walls and membranes of haloarchaea are easy to lyse by osmotic shock with the use of distilled water, as demonstrated in this work. This allows for the recovery of PHBV granules with the use of fewer chemicals and in a much easier and more economical way [6,10,14]. Particularly, most haloarchaea, including *Hfx. mediterranei*, can synthesize the copolymer PHBV even without any external precursor, as confirmed by ATR–FTIR analysis in this work (Figure 5 and Table 1); moreover, they contribute to reducing the production costs [44,45]. Furthermore, raw materials like brines could even be used to prepare culture media for these strains, thus contributing to circular economy processes [46]. These advantages constitute an important aspect to consider when designing large-scale biotechnological approaches that allow for the reduction of production costs. Consequently, extremophiles have recently been identified as one of the most promising microorganisms to produce PHA, which reduce production costs [47].

Studies of screening methods for the presence and characterization of PHAs in archaea, and especially in haloarchaea, using fluorescent dyes such as Nile red are scarce. Like bacteria, archaea possess a cell wall that protects the cell from the environment, though their membranes differ from those of bacteria and other organisms in different aspects. Archaea cell walls possess ether linkages instead of ester linkages; they are composed of regularly branched phytanyl and biphytanyl chains instead of fatty acyl chains; the glycerol linkage between the phospholipid head and the side chain is in the L-isomeric form, while bacteria and eukaryotes have the D-isomeric form, and proteins also tend to be rich in surface-exposed negatively charged amino acids [33,34,35]. These properties contribute to the greater chemical stability of archaeal envelopes, but also to the low permeability of archaeal membranes, hindering staining. Most haloarchaea such as *Hfx. mediterranei* and *Hfx. volcanii* have a proteinaceous S-layer anchored to the microorganism’s surface that functions as a protective coat, molecular sieve, molecule and ion traps, as well as perform roles in surface recognition and cell shape maintenance [48]. The S-layer constitute the only cell envelope structure in haloarchaea, with the exception of halococci and certain strains of *Haloquadratum walsbyi*. The S-layer lattice type in halophilic archaea is hexagonal, its center-to-center spacing distance value is between 12 and 16 nm (similar to that detected in most methanogens), and it presents an acidic amino acid composition and glycosylation [33]. The exposed glycan chains influence the surface roughness of the cell on the nanometer scale and cause the formation of a lubrication hydration layer. The presence of the S-layer as the only cell envelope structure in haloarchaea may facilitate the incorporation of dyes as compared to other archaea with an S-layer in addition to the cell wall. However, haloarchaea exhibit optimal growth in salt concentrations approaching the saturation point; the high ionic strength of the medium is inappropriate for conventional antibodies and dyes, and salt crystals interfere with microscopic observation of cells, especially when no staining method is used as demonstrated in the phase contrast images of *Hfx. mediterranei*. In addition, the high intracellular content of potassium ions to counterbalance the high sodium concentration may complicate staining [49].

Most of the published works use transmission electron microscopy (TEM) to visualize PHA granules in haloarchaea [9,10,23]. As shown in Figure 3, TEM is an excellent tool for imaging PHA granules and characterizing their size and shape [27]. However, it requires working with fixed cells, and is a time-consuming technique comprising many steps (fixation with several fixation agents, dehydration, embedding, and ultrathin section cutting) that require highly experienced staff, in addition to the higher cost of the technique as compared to fluorescence staining. Legat et al. (2010) reported for the first time the use of three dyes (Sudan black, Nile blue A, and Nile red) for the easy and quick identification of PHA-producing haloarchaea, thus demonstrating the permeability of the S-layer and the cell wall to these dyes and allowing the identification of a new PHA-producing *Halococcus* species [31]. Sudan black B and Nile blue dyes were employed to stain fixed cells, and PHA granules were subsequently observed by optical and fluorescence microscopy, respectively. They also observed a diffuse staining, which roughly delineated the cell morphology and could be attributed to other lipid structures such as membranes or haloarchaeal S-layers [34]. Nile red staining was used for the identification of PHA-producing colonies growing on agar plates by UV light exposure, showing the presence of fluorescent colonies in many haloarchaeal species [31]. Mahansaria et al. (2015) also reported the use of Nile red in the staining of growing colonies for the identification of PHA producers among halobacteria and haloarchaea, and in the visualization of fluorescence colonies with a UV transilluminator in addition to a polymerase chain reaction-based screening method, through the amplification of a conserved region of the Class III PHA synthase (*phaC*) gene [32]. Both works reported the use of Nile red to identify PHA-producing microorganisms; stained cells were not observed with fluorescence microscopy, but with a UV transilluminator. Due to the lipophilic nature of this dye, it can bind to other lipid inclusions, membranes, or haloarchaeal cell envelopes and falsely depict the organism such as a PHA producer upon its exposure to UV light.

Nile red staining can be performed in living cells under growing conditions, allowing for the visualization of PHAs as fluorescent red granules. In haloarchaea, the presence of salt crystals in the medium, even after washing the cells, hinders the identification of cells by confocal microscopy and the co-localization of Nile red-stained PHA granules inside the cytoplasm of cells, as shown in Figure 1 and Figure 2. Moreover, due to its lipophilic nature, Nile red can bind unspecifically to other cell structures [31], making it important to use an additional fluorescent dye to corroborate the localization of PHA granules. In this study, we are presented with the first visualization of PHA granules in unfixed cells by confocal microscopy, as well as the use of a second fluorescence staining with the fluorescent cyanine dye SYBR Green [50] in providing a rapid and easy method for the identification of PHA granules (Figure 4). This double-staining method with Nile red and SYBR Green in *Hfx. mediterranei* is proven to be an appropriate way to co-localize PHA granules inside the cytoplasm of cells by confocal microscopy, merging both channels of fluorescence and allowing the analysis of PHA granule size (Figure 4). This method demonstrates that these dyes are compatible with media of high ionic strength. This involves a faster, simpler, and more economical procedure for identifying PHAs. The promising future of these biopolymers sheds light on a global problematic, making the study of their potential indispensable in developing more sustainable production methods.

## 5. Conclusions

This work optimizes a new, quick, and cheap method that uses Nile red and SYBR Green to detect PHA production by haloarchaea and to quantify the mean size of PHA granules in living cells, thus demonstrating the utility of this method in the Archaea domain. This involves a simpler, more affordable, and faster procedure for identifying PHAs during a period wherein the urge to replace conventional oil-based plastics is of international interest. Consequently, this aids in boosting the development of archaeal cell factories as promising and sustainable biotechnological platforms for PHA production.

## Figures and Tables

**Table 1:** CDW, PHBV production and yield obtained after 96 h of fermentation in a shaken flask. SD = standard deviation. All data are expressed as the mean ± SD of three independent experiments.

Strain	Carbon Source	CDW (g/L)	PHA (g/L)	Yield (PHA/CDW) g g^−1^
*Haloferax mediterranei* R-4 (ATCC33500)	Glucose 10g/L	2.31 ± 0.26	0.198 ± 0.06	0.084 ± 0.02

## Data Availability

Not applicable.
